# Proenkephalin A for assessing kidney integrity and guiding KRT liberation decisions in critically ill patients

**DOI:** 10.1016/j.aicoj.2026.100113

**Published:** 2026-07-21

**Authors:** David C. Gabriel, Philipp Sauer, Rebecca Happel, Julia Grenz, Louise Benning, Florian Kälble, Florian Uhle, Felix C.F. Schmitt, Uta Merle, David Czock, Markus Verch, Matthias Karck, Mascha O. Fiedler, Martin Zeier, Christian Morath, Markus A. Weigand, Christian Nusshag

**Affiliations:** a Department of Nephrology, Heidelberg University Hospital, Medical Faculty, Heidelberg University, Heidelberg, Germany; b SphingoTec GmbH, Henningsdorf/Berlin, Germany; c Department of Anesthesiology, Heidelberg University Hospital, Medical Faculty, Heidelberg University, Heidelberg, Germany; d Department of Gastroenterology, Heidelberg University Hospital, Medical Faculty, Heidelberg University, Heidelberg, Germany; e Department of Clinical Pharmacology and Pharmacoepidemiology, Heidelberg University Hospital, Medical Faculty, Heidelberg University, Heidelberg, Germany; f Department of Cardiac Surgery, Heidelberg University Hospital, Medical Faculty, Heidelberg University, Heidelberg, Germany; g Department of Nephrology and Hypertension, Klinikum Nuremberg, Paracelsus Medical University, Nuremberg, Germany

**Keywords:** Proenkephalin A, PENK Acute Kidney Injury, Disease Staging, Kidney Replacement Therapy Liberation, Functional Kidney Integrity, Biomarker Guidance

## Abstract

**Background:**

Utility of serum creatinine (SCr) and urine output (UO) is limited for real-time assessments of functional integrity and guiding kidney replacement therapy (KRT) decisions in acute kidney injury (AKI). Proenkephalin A 119–159 (PENK) may overcome these limitations. We evaluate PENK’s use for AKI staging, assessment of residual kidney function during KRT, and assessment of liberation failure.

**Methods:**

Prospective, real-world study in 1,436 critically ill patients at Heidelberg University Hospital, including a subgroup of 138 patients receiving KRT for liberation analyses. Plasma PENK was measured from admission to discharge. AKI was defined by KDIGO criteria; biomarker kinetic, ROC analyses and binary logistic regression models were performed. KRT liberation was considered successful if no re-initiation occurred within >5 days.

**Results:**

Of 1,436 patients, 621 (43.2%) developed AKI. Acute KRT was required in 12.6%, 40.9% of whom were successfully liberated, 24.3% failed, and 31.5% underwent no liberation attempt (3.3% lost to follow up). PENK rose with AKI stages and, unlike SCr/UO, distinguished stage 3 AKI with versus without acute KRT requirements, and differentiated acute from chronic KRT patients even under ongoing KRT. From KRT start, mean PENK levels increased progressively whereas SCr decreased under treatment. Later, PENK declined around successful liberation, with lower values compared to cases with liberation failure. PENK was the strongest, independent predictor of KRT liberation failure, outperforming SCr. A PENK cut-off of ≥250 pmol/L provided >90% specificity for liberation failure. Combining PENK and UO, further improved risk stratification.

**Conclusions:**

PENK may reflect kidney integrity independent of KRT, enhance residual kidney function assessment, and may aid prediction of KRT liberation failure, warranting multicenter validation.

## Background

Acute kidney injury (AKI) is a frequent and serious complication in critically ill patients, with an incidence of up to 57% in intensive care units (ICUs) [1,2]. It is associated with substantial morbidity and mortality, particularly among those requiring kidney replacement therapy (KRT) [2,3]. Survivors of AKI-related critical illness often show greater declines in health-related quality of life and face increased risk of chronic kidney disease (CKD) and kidney failure, contributing to long-term healthcare and societal burden [[4], [5], [6]].

AKI is not a single disease entity but a complex syndrome resulting from diverse intra- and extra-renal insults [2,7]. According to Kidney Disease: Improving Global Outcomes (KDIGO) guidelines, diagnosis is still based on changes in serum creatinine (SCr) and urine output (UO) [8]. However, both parameters have major limitations in sensitivity, specificity, and reliability, particularly in critically ill patients and those undergoing KRT [9,10]. UO, while sensitive, lacks specificity and may decline with volume depletion without indicating intrinsic kidney injury [11]. It also correlates poorly with glomerular filtration rate (GFR), its recovery, or residual kidney function. SCr, on the other hand, is a delayed marker influenced by muscle mass, fluid shifts, and extracorporeal clearance, rendering it especially unreliable during KRT [8,10]. Together, these shortcomings highlight the need for biomarkers that provide more accurate assessment of functional kidney integrity, particularly under non–steady-state conditions and during KRT.

Further, a key clinical challenge remains when to initiate or discontinue KRT. Current practice relies largely on clinical judgment and surrogate markers rather than standardized criteria [12]. Liberation from KRT is usually guided by UO, volume status, and SCr kinetics – especially in intermittent hemodialysis (IHD) [13] – but decisions remain highly subjective, with reported success rates ranging from between 20% and 54% [14,15].

Proenkephalin A 119–159 (PENK) has emerged as a novel functional biomarker. With a molecular weight of 4.5 kDa, PENK is a stable peptide derived from the same precursor as endogenous enkephalins [[16], [17], [18]]. It is non–protein-bound, and minimally affected by age, sex, or systemic inflammation [17,19,20]. Recent studies suggest PENK may outperform conventional markers in predicting successful KRT liberation [[21], [22], [23]]. However, current evidence is limited by the lack of direct comparisons with established standards (SCr and UO), small sample sizes, post hoc designs, and insufficient longitudinal data across AKI severity stages, KRT modalities, and recovery phases [17,[21], [22], [23]]. In particular, PENK’s temporal dynamics around key clinical events – such as peak AKI severity, KRT initiation and discontinuation – remain insufficiently explored [24].

In this observational, exploratory study across three ICUs at Heidelberg University Hospital, we evaluated the longitudinal utility of PENK in a real-world critical care setting. We hypothesized that PENK refines AKI severity assessment and serves as a marker of kidney functional integrity during KRT, enabling early identification of patients at risk for liberation failure.

## Methods

### Study design

This exploratory, observational, real-world study was conducted at Heidelberg University Hospital between November 2021 and July 2023. Plasma PENK levels were measured on weekdays (Monday–Friday) in 1,436 critically ill adults (≥18 years) admitted to three ICUs from admission to discharge. Analyses of PENK across AKI stages were performed in the overall cohort, whereas analyses of KRT exposure and KRT liberation were conducted in a subgroup of 138 patients receiving KRT. AKI staging was based on daily SCr and UO measurements according to KDIGO criteria. Analyses involving KRT initiation, discontinuation, and liberation were restricted to patients with paired PENK and SCr measurements available at the respective clinical time points. Accordingly, specific analyses were conducted only in patient subsets defined by the availability of paired biomarker measurements and the occurrence of relevant clinical events. PENK measurements were integrated into routine morning blood sampling and analyzed by the accredited Central Laboratory of Heidelberg University Hospital using the sphingotest® penKid® immunoassay (SphingoTec GmbH, Hennigsdorf, Germany) [25]. The study was conducted as part of the PARTICIPATE trial [26], which investigated the diagnostic and clinical utility of PENK in various clinical contexts such as critical illness and after kidney transplantation. Ethical approval was obtained from the Ethics Committee of the University of Heidelberg (S-714/2021), and the study was registered in the German Clinical Trials Register (DRKS00026776) before inclusion of the first study participant. All procedures were carried out in accordance with the Declaration of Helsinki. Patient consent was waived as PENK assessment was integrated into routine diagnostics in all ICU patients, imposing no additional burden. PENK kinetics were analyzed in relation to AKI severity and KRT exposure and compared with standard clinical parameters.

### Data collection and kidney replacement therapy characteristics

At ICU admission, demographic data (age, sex, BMI), comorbidities, sepsis/septic shock status, admission type, organ dysfunction, and severity scores (Sequential Organ Failure Assessment [SOFA] and Acute Physiology, Simplified Acute Physiology II Score [SAPS] and Acute Physiology And Chronic Health Evaluation II Score [APACHE II]) were recorded. Additional variables included laboratory values, vasopressor use, 24 h urine output and dosage of loop diuretics prior last KRT, modality of organ support, ICU length of stay, and in-hospital mortality. Continuous kidney replacement therapy (CKRT) was performed with Prismaflex/Prismax (Baxter, Illinois, USA) using M150 membranes and regional citrate anticoagulation; pre-dilution 1000−1300 mL/h (citrate dose-dependent, Prismocitrate, Baxter), blood flow 100−120 mL/min, dialysate 1000−1250 mL/h (Phoxillium, Baxter), and post-filter substitution 500 mL/h (Phoxillium, Baxter). Sustained low-efficiency dialysis (SLED) and IHD were performed with the Genius system (Fresenius Medical Care, Hessen, Germany) using FX50 membranes (Fresenius). For SLED, blood flow was 120–180 mL/min and dialysate 60–90 mL/min; for IHD, both blood and dialysate flows were 180–250 mL/min. Unfractionated heparin was used for anticoagulation during SLED and IHD. The choice of KRT modality was based on the patient's clinical condition and hemodynamic stability rather than the presence or absence of pre-existing dialysis-dependent CKD.

### Endpoint definitions

AKI was defined according to KDIGO urine output and SCr criteria (mild AKI: KDIGO stage 1; moderate AKI: KDIGO stage 2; severe AKI: KDIGO stage 3). Baseline SCr was determined as described in the Supplementary Material. Patients were classified by their maximum AKI stage within 7 days of ICU admission. CKD was defined as eGFR (CKD-EPI) <60 mL/min/1.73 m^2^, calculated from baseline SCr. KRT initiation followed a joint decision by the attending intensivist and senior nephrologist according to an internal protocol informed by the AKIKI I and II trials [27,28], as illustrated in Supplementary Table S1. Liberation was reassessed daily and jointly decided by the treating intensivist and nephrologist. For standardized analysis, the start of a liberation trial was defined as a ≥48-h KRT-free interval and deemed successful if KRT was not resumed for ≥5 days.

### Statistical analysis

Continuous variables are presented as medians with interquartile ranges (IQR), categorical variables as numbers and percentages. Multiple group comparisons for continuous data were performed using Kruskal–Wallis test with Dunn’s post hoc analysis; pairwise comparison was done by Mann Whitney test; categorical data were analyzed using Pearson’s Chi-squared test. Diagnostic performance was evaluated by receiver-operating characteristic (ROC) analysis, with AUROC used to compare biomarker accuracy. Furthermore, to adjust for potential confounders relevant to KRT liberation, a multivariate regression model for the endpoint KRT liberation failure was performed. The latter model included age, gender, SOFA score, vasopressor support, SCr at last KRT, 24 h UO prior last KRT < 500 mL, pre-existing liver cirrhosis, diuretic dose 24 prior last KRT and the PENK threshold ≥250 pmol/L as covariates.

For all analyses, only patients with available paired PENK or SCr values were included. All tests were two-sided with a significance level of P < 0.05. Statistical analyses and figures were generated using GraphPad Prism (v9.2.0) and SPSS (v30.0.0); boxplot whiskers indicate the 10th to 90th percentiles.

## Results

### Study population

**Fig. 1:**
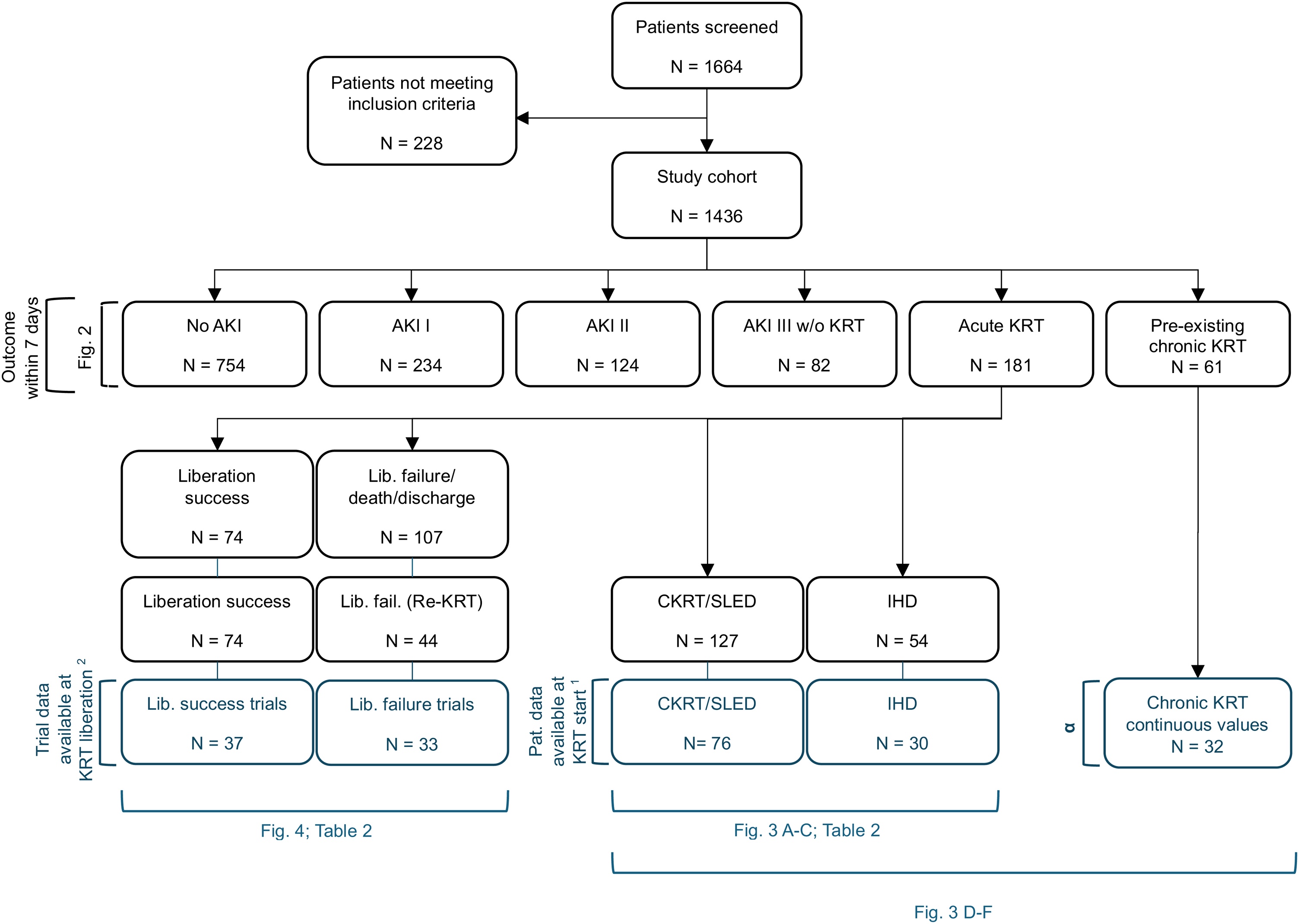
Flow chart of study design and data analysis. AKI acute kidney injury, KRT kidney replacement therapy, CKRT continuous veno-venous hemodiafiltration, SLED sustained low-efficiency dialysis (Genius-System), IHD including intermittent hemodialysis, acute KRT patients with acute need for KRT during ICU stay, pre-existing chronic KRT patients with KRT dependency prior ICU admission. ^1^Data available at KRT start, ^2^data available at the time of KRT liberation, ^α^chronic KRT patients with continuous PENK values eligible for comparison with acute KRT patients.

Between November 2021 and July 2023, 1,664 patients were admitted to three ICUs at Heidelberg University Hospital; 1,436 met eligibility criteria (Fig. 1), while 228 were excluded for missing PENK measurements or age <18 years. Within 7 days post-enrolment, 621 patients (43.2%) developed AKI: 234 (16.3%) mild, 124 (8.7%) moderate, and 263 (18.3%) severe. Acute KRT was required in 181 patients (12.6%); 70.2% received continuous KRT or SLED and 29.8% IHD. An additional 61 patients (4.3%) were admitted with pre-existing kidney failure and KRT-dependency (chronic KRT).

Compared with patients not requiring KRT, acute KRT patients had higher baseline and admission SCr, lower UO, and greater disease severity scores (SOFA, SAPS II, APACHE II), although mean baseline SCr remained within the normal range within both groups. Acute KRT patients showed lower rates of hypertension and coronary artery disease but more liver cirrhosis and malignancy (Table 1). Clinically, they more often underwent emergency surgery, and had higher rates of sepsis, septic shock, vasopressor use, and mechanical ventilation. They also had higher maximum SCr and PENK levels, longer ICU stays, and higher mortality (Table 1).

**Table 1:** Baseline characteristics and outcomes.

Demographics	Study cohort w/o KRT patients (N = 1194)	Acute KRT Patients (N = 181)	Chronic KRT patients (N = 61)	Study cohort w/o KRT vs. acute KRT (p-value)	Acute KRT vs. chronic KRT (p-value)
Age – yr	66 (58−74)	65 (57−73)	66 (56−73)	0.228	0.799
Female sex – no. (%)	329 (27.6)	63 (34.8)	19 (31.2)	0.114	0.602
Body-Mass-Index – kg/m^2^	26.5 (23.8−30.1)	26.3 (23.1−30.9)	24.3 (21.7−30.6)	0.068	0.049
Baseline serum creatinine prior ICU (w/o ESKD) – mg/dL (IQR)α	0.88 (0.73−1.07)	1.02 (0.79−1.34)	–	<0.001	–
Kidney parameters and ICU Scores on admission					
PENK ICU – pmol/L (IQR)	48.8 (37.3−68.5)	95.3 (59.0−138.4)	416.9 (252.3−718.6)	<0.001	<0.001
Serum creatinine	0.91 (0.73−1.22)	1.74 (1.15−2.49)	4.11 (2.22−6.59)	<0.001	<0.001
Urea – mg/dL (IQR)	36 (28−50)	67 (46−104)	68 (51−90)	<0.001	0.847
Oliguria or anuria – no. (%)ꝉ	30 (2.5)	74 (40.9)	31 (50.8)	<0.001	0.176
SOFA scoreβ	6 (3−8)	13 (10−15)	10 (6−13)	<0.001	<0.001
SAPS II valueγ	33 (26−44)	66 (51−78)	50 (39−70)	<0.001	<0.001
APACHE II scoreδ	17 (14−22)	29 (22−35)	25 (20−32)	<0.001	0.017
Preexisting comorbidities – no. (%)					
Chronic kidney disease (eGFR <60 mL/min/1,73m^2^)	202 (16.9)	61 (33.7)	61 (100)	0.011	<0.001
Pre-existing kidney failure	0 (0.0)	0 (0.0)	61 (100)	0.999	<0.001
Arterial Hypertension	883 (74.0)	124 (68.5)	55 (90.2)	<0.001	0.001
Coronary artery disease	759 (63.6)	66 (36.5)	36 (59.0)	<0.001	<0.002
Congestive heart failure	400 (33.5)	52 (28.7)	19 (31.2)	0.203	0.712
Aortic aneurysm	181 (15.2)	24 (13.3)	4 (6.6)	0.504	0.157
Diabetes mellitus	365 (30.6)	48 (26.5)	27 (44.3)	0.268	0.0096
Liver cirrhosis	63 (5.3)	21 (11.6)	5 (8.2)	0.001	0.458
Malignant disease	156 (13.1)	40 (22.1)	8 (13.1)	0.001	0.128
Admission category – no. (%)					
Elective surgery	644 (53.9)	43 (23.7)	18 (29.5)	<0.001	0.371
Emergency surgery	473 (39.6)	110 (60.8)	35 (57.4)	<0.001	0.640
Medical	77 (6.5)	28 (15.5)	8 (13.1)	0.190	0.655
Outcomes					
Mechanical ventilation – no. (%)	899 (75.3)	162 (89.5)	41 (67.2)	<0.001	<0.001
Sepsis – no. (%)	89 (7.5)	89 (49.2)	21 (34.4)	<0.001	0.046
Septic shock – no. (%)	77 (6.5)	66 (36.5)	17 (27.9)	<0.001	0.221
Vasopressor support – no. (%)	799 (66.9)	162 (89.5)	47 (77.1)	<0.001	0.014
Cumulative fluid balance – mL/ first 24 h (IQR)	1395 (500−2496)	1850 (702−3854)	1362 (341−2550)	0.576	0.782
Maximum serum creatinine – mg/dL (IQR)	1.06 (0.85−1.55)	3.33 (2.47−4.65)	5.13 (3.58−6.98)	<0.001	<0.001
Maximum PENK – pmol/L (IQR)	52.9 (40.0.−77.0)	219.0 (133.6−358.2)	547.9 (351.0−739.3)	<0.001	<0.001
Length of ICU stay – days	3 (2−8)	26 (13−47)	9 (5−20)	<0.001	<0.001
Hospital mortality (%)	48 (4.0)	75 (41.4)	16 (26.2)	<0.001	0.034

ESKD end-stage kidney disease, IQR interquartile range, KRT kidney replacement therapy, PENK proenkephalin A 119–159.

*Values are represented as median plus (interquartile range). Listed are data from adult patients who have at least one PENK value. AKI denotes acute kidney injury, eGFR CKD-Epi estimated glomerular filtration rate chronic kidney disease epidemiology collaboration, IQR interquartile range.

α Baseline serum creatinine was acquired for all patients without end-stage renal disease.

β Scores on the Sequential Organ Failure Assessment (SOFA) range from 0 to 24, with larger values indicating greater disease severity and mortality.

γ Results for the Simplified Acute Physiology Score (SAPS) II range from 0 to 163, with higher scores indicating greater disease severity and mortality.

δ Results for the Acute Physiology And Chronic Health Evaluation (APACHE) II score range from 0 to 71, with higher scores indicating greater disease severity and mortality.

ꝉ Oliguria was defined as urinary output <500 ml within a 24-h period.

Chronic KRT patients had demographics like those with acute KRT but lower disease severity scores. They more often suffered from hypertension, coronary artery disease, and diabetes, while other comorbidities were comparable. Compared with acute KRT patients, they required less mechanical ventilation, had lower rates of sepsis and vasopressor support, shorter ICU stays, and lower mortality (Table 1).

### Trajectory and characteristics of acute kidney replacement therapy

Of 181 patients requiring acute KRT, 74 (40.9%) achieved successful liberation. In the remaining 107 patients (59.1 %), 44 (24.3%) had explicit liberation failure requiring KRT re-initiation (Re-KRT), 57 (31.5%) never underwent a liberation attempt, and 6 (3.3%) were discharged within five days of liberation trial initiation and lost to follow-up (Fig. 1, Table 2). The main indication for first KRT was hypervolemia, followed by uremia and hyperkalemia, with metabolic acidosis less frequent (Table 2). For Re-KRT, hypervolemia and uremia remained predominant, while hyperkalemia and acidosis were less common. CKRT/SLED was the preferred initial modality as well as more likely applied during Re-KRT. Critical parameters leading to KRT initiation are summarized in Supplementary Table S1.

**Table 2:** Procedural characteristics of acute KRT.

Total patients – no.	181
KRT days performed per patient – days (IQR)	14 (5−37)
Ultrafiltration volume per day – mL (IQR)	2100 (1090−3141)
KRT indication – no. (%)	
Hypervolemia	109 (60.2)
Uremia	32 (17.7)
Hyperkalemia	31 (17.1)
Metabolic acidosis	9 (4.9)
First KRT modality – no. (%)	
CKRT/SLED	126 (69.6)
IHD	55 (30.4)
Patients with KRT liberation success – no. (%) ꝉꝉ	**74 (40.9)**
Patients with KRT liberation failure – no. (%)	**44 (24.3)**
Patients with no liberation trial until discharge or death – no. (%)	**57 (31.5)**
Lost to follow up – no. (%)	**6 (3.3)**
Indication Re-KRT liberation failure (N = 44) – no. (%)	
Hypervolemia	26 (59.1)
Uremia	8 (18.2)
Hyperkalemia	7 (15.9)
Metabolic acidosis	3 (6.8)
Re-KRT modality (N = 44) – no. (%)	
CKRT/SLED	28 (63.6)
IHD	16 (36.4)

CKRT Continuous kidney replacement therapy, IHD intermittent hemodialysis, IQR interquartile range, KRT kidney replacement therapy, PENK proenkephalin A 119–159, SLED Sustained low-efficiency dialysis.

*Values are represented as median plus (interquartile range). Listed are data from adult patients who have at least one PENK value. AKI denotes acute kidney injury, eGFR CKD-Epi estimated glomerular filtration rate chronic kidney disease epidemiology collaboration, IQR interquartile range.

ꝉꝉ Successful liberation from KRT was defined as a period of at least 5 consecutive days without need for KRT.

### Plasma levels of PENK and SCr stratified by AKI severity

**Fig. 2:**
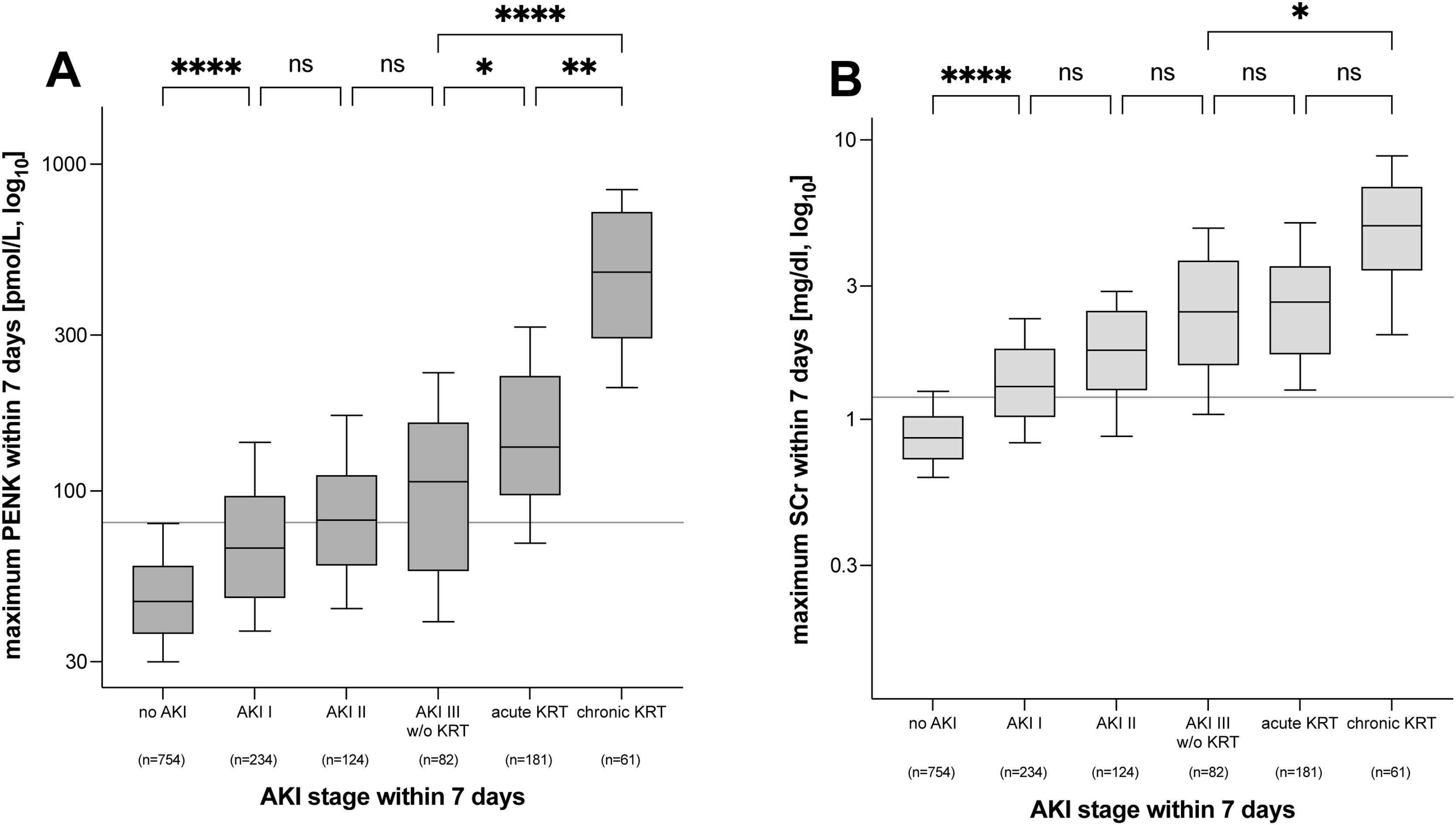
Maximum PENK (A) and SCr levels (B) stratified by AKI stage within 7 days of admission. AKI acute kidney injury, PENK proenkephalin, KRT kidney replacement therapy, SCr serum creatinine. Gray lines indicate upper normal range. Statistical significance is indicated as follows: ns, *P* ≥ 0.05; *P* < 0.05 (*); *P* < 0.01 (**); *P* < 0.001 (***); *P* < 0.0001 (****).

Within seven days of ICU admission, patients without AKI had lower median peak PENK (45.9 pmol/L) and SCr (0.86 mg/dL) levels than those with mild AKI (66.8 pmol/L, 1.31 mg/dL; Fig. 2). In moderate and severe AKI without KRT, both biomarkers increased in a stage-dependent manner without reaching statistical significance. Compared with severe AKI without KRT, PENK showed significantly higher levels in patients with acute KRT and chronic KRT, whereas SCr and UO did not differ (Fig. 2).

### Biomarker trajectory under KRT conditions

**Fig. 3:**
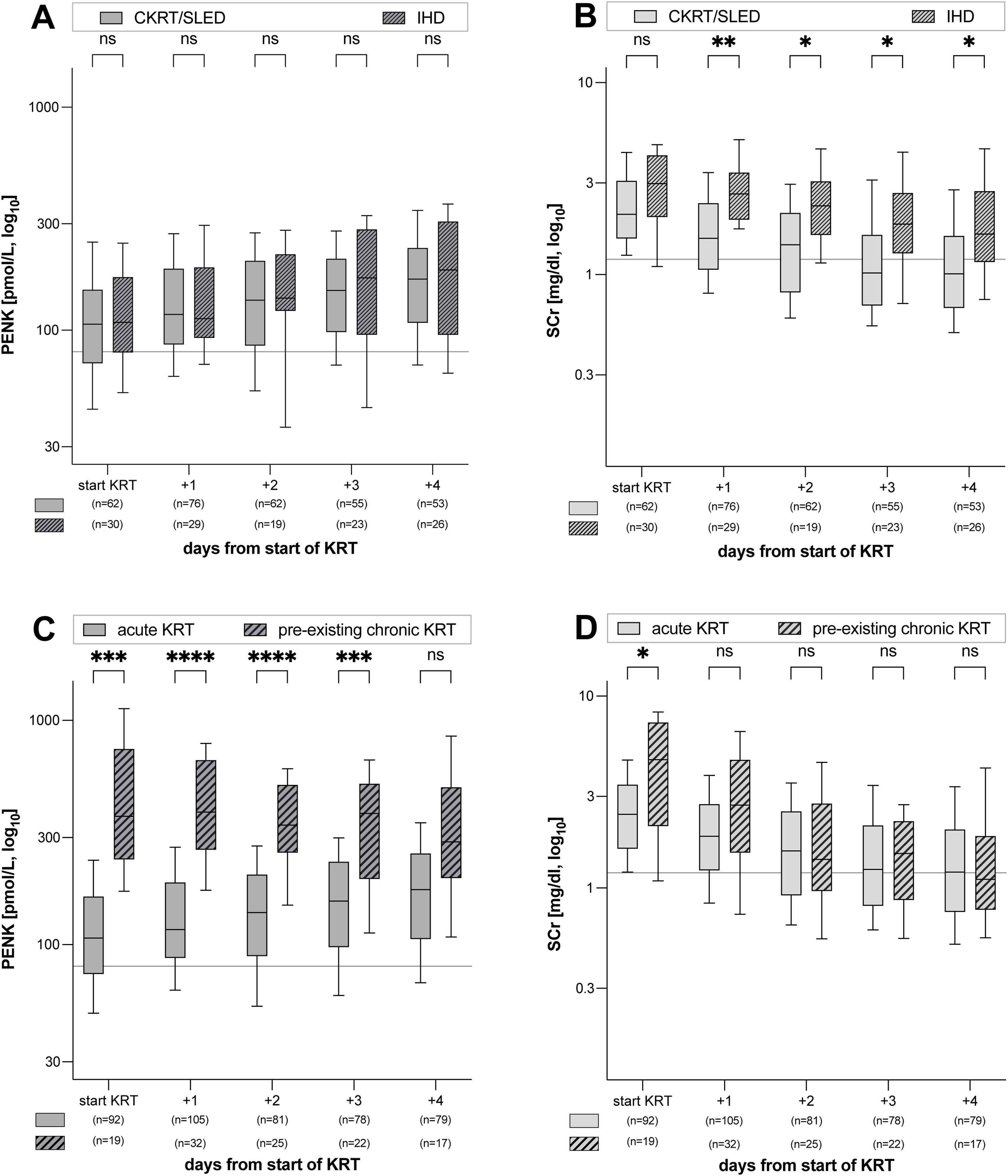
PENK and SCr levels stratified by KRT modality and acute versus chronic KRT. A + B timely matched course of PENK and SCr in relation to the start of kidney replacement therapy stratified by KRT modalities. C + D timely matched course of PENK and SCr in relation to start of kidney replacement therapy stratified comparing acute KRT versus pre-existing chronic KRT (N = 138). CKRT continuous kidney replacement therapy, IHD intermittent hemodialysis, PENK proenkephalin, KRT kidney replacement therapy, SCr serum creatinine, SLED sustained low-efficiency dialysis (Genius). Gray lines indicate upper normal range. Statistical significance is indicated as follows: ns, *P* ≥  0.05; *P* < 0.05 (*); *P* < 0.01 (**); *P* < 0.001 (***); *P* < 0.0001 (****).

When stratified by KRT modality (CKRT/SLED vs. IHD), longitudinal PENK levels remained elevated with an upward trend during KRT, irrespective of modality. Median levels were 106.4 pmol/L for CKRT/SLED and 108.5 pmol/L for IHD at initiation, rising to 176.0 and 287.4 pmol/L by day 4 (NS; Fig. 3A), respectively. In contrast, SCr declined significantly in both groups, more rapidly with CKRT/SLED (from day 1; Fig. 3B). Similar trends were observed for biomarker levels in individual patients across KRT modalities (Supplementary Figure S1). Further, PENK remained elevated in patients without any liberation attempts, comparable to those with failed liberation, and tended to be higher than in successfully liberated patients (Supplementary Figure S2).

To assess PENK as a marker of residual kidney integrity during KRT, patients with acute vs. chronic KRT were compared (Fig. 3C+D). In acute KRT, PENK rose after initiation, whereas in chronic KRT it was already strongly elevated and largely stable. During the first 3 days, PENK was significantly higher in chronic KRT, clearly distinguishing both groups despite KRT initiation (Fig. 3D). By contrast, SCr differed initially but converged over time (Fig. 3E).

### PENK indicating KRT liberation failure

**Fig. 4:**
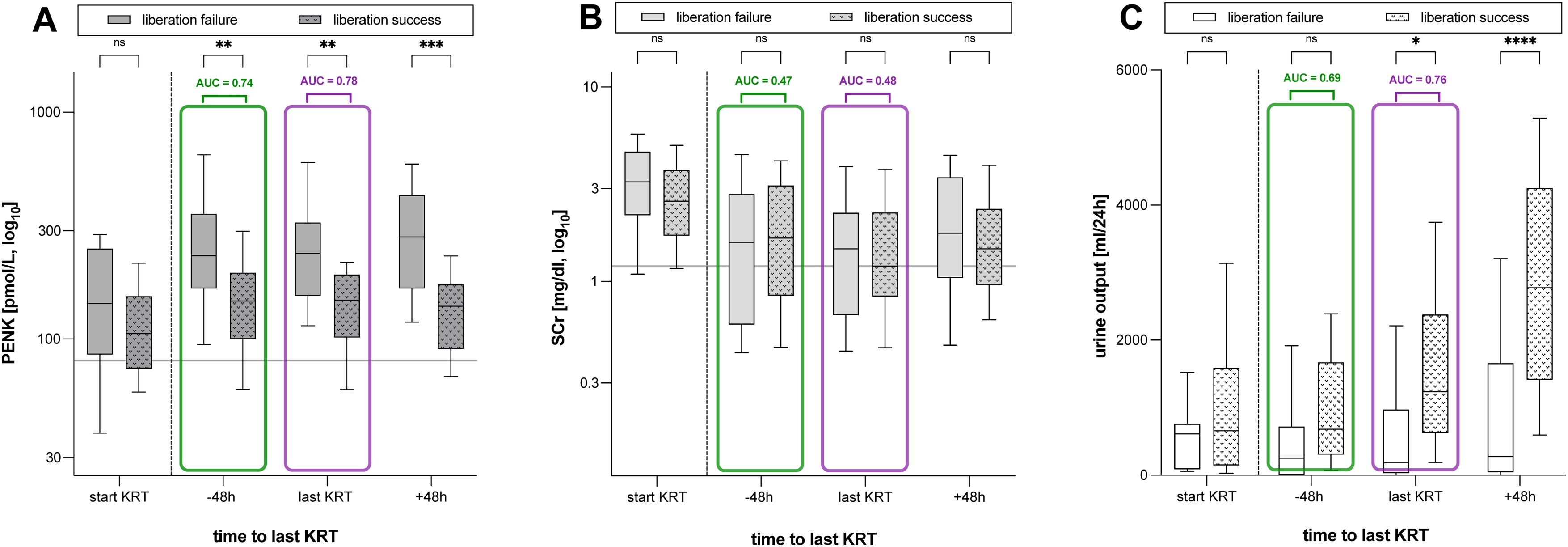
Biomarker kinetics and receiver-operating characteristic (ROC) analysis of PENK to differentiate liberation failure (N = 33) from KRT liberation success (N = 37) in comparison to SCr and 24 h urine output (N = 70). AUC area under the curve, PENK proenkephalin, KRT kidney replacement therapy, SCr serum creatinine. Gray lines indicate upper normal range. Statistical significance is indicated as follows: ns, *P* ≥ 0.05; *P* < 0.05 (*); *P* < 0.01 (**); *P* < 0.001 (***); *P* < 0.0001 (****).

We evaluated whether PENK offers additive diagnostic information beyond SCr and UO in identifying failed KRT liberation attempts. Thus, all three parameters were analyzed relative to the timing of each patient's liberation attempt and aligned accordingly (Fig. 4). Baseline characteristics stratified by successful versus failed liberation are provided in the supplemental material (Table S2). PENK levels were significantly higher and OU lower in patients with failed versus successful KRT liberation. At the time of the last KRT session, median PENK levels were 238.8 pmol/L vs. 148.4 pmol/L (p < 0.001), and 48 h prior, 233.0 pmol/L vs. 147.1 pmol/L (p < 0.001) (Fig. 4A) in patients with and without KRT liberation failure, respectively. In contrast, SCr showed no significant differences: 1.47 mg/dL vs. 1.20 mg/dL at last session (p = 0.746), and 1.59 mg/dL vs. 1.67 mg/dL at 48 h prior (p = 0.680) (Fig. 4B). UO was not significantly different between groups 48 h prior last KRT session, with median values of 250.0 vs. 680.0 mL/24 h (p = 0.06) (Fig. 4C).

To assess predictive performance for liberation failure AUC-ROC analyses were performed. At the time of the last KRT session, PENK (AUC-ROC: 0.78; 95% CI: 0.66–0.89) and UO (0.76; 95% CI: 0.65–0.88) showed superior predictive accuracy, outperforming SCr (0.48; 95% CI: 0.34–0.62). At 48 h prior, PENK remained the best predictor (AUC-ROC: 0.74; 95% CI: 0.62–0.86), whereas SCr (0.47; 95% CI: 0.33–0.61) and UO (0.69; 95% CI: 0.56–0.81) showed poorer performance. Combination of PENK and UO further improved AUC-ROC for liberation failure (last KRT: 0.83; 95% CI: 0.73–0.93; 48 h prior last KRT: 0.78; 95% CI: 0.67–0.89). To establish a threshold for ruling in liberation failure with >90% specificity, a PENK cut-off ≥250 pmol/L yielded 94.6% specificity and 48.5% sensitivity at the time of last KRT, with a PPV of 88.8% and NPV of 67.3%. At 48 h prior, the same cut-off yielded 89.2% specificity and 45.5% sensitivity (95% CI: 0.24–0.51), with a PPV of 78.9% and NPV of 64.7%.

**Fig. 5:**
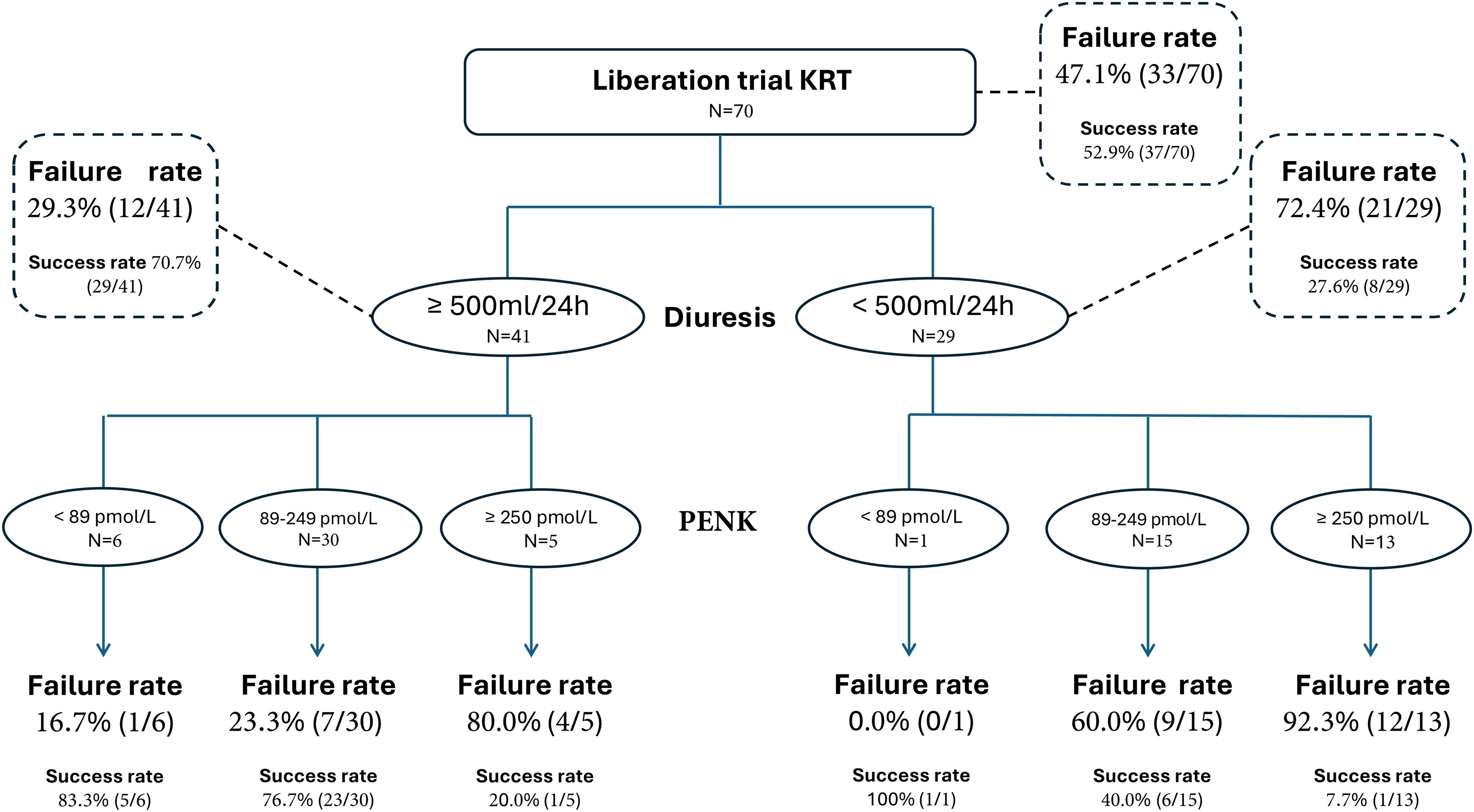
Decision tree integrating 24-h urine output (UO) and PENK for prediction of CKRT liberation outcomes (N = 70). Patients were first stratified by UO ≥ 500 mL/24 h or <500 mL/24 h, followed by PENK categories (<89 pmol/L, 89–249 pmol/L, ≥250 pmol/L). Failure and success rates of liberation trials are shown for each subgroup. KRT kidney replacement therapy, PENK proenkephalin, UO urine output.

Given the complexity of CKRT liberation decisions, we further constructed a decision tree integrating 24 h UO prior liberation together with PENK for outcome assessment (Fig. 5). PENK stratification [<89 pmol/L (normal range), 89−249 pmol/L (intermediate group), and ≥250 pmol/L (high)] markedly refined risk estimates. Among patients with UO ≥ 500 ml/24 h, failure rates were 16.7%, 23.3%, and 80.0% across the respective subgroups. In patients with UO < 500 ml/24 h, failure rates were 0%, 60.0%, and 92.3%.

Lastly, given the critical role of loop diuretics and other potential confounders in facilitating KRT liberation and influencing UO thresholds, we constructed a multivariable model for KRT liberation failure adjusting for age, gender, SOFA score, vasopressor support, SCr at last KRT, 24 h UO prior last KRT < 500 mL, pre-existing liver cirrhosis and loop diuretic dose 24 prior last KRT (Table 3). In this model, PENK levels ≥250 pmol/L remained the strongest independent predictor together with liver cirrhosis for liberation failure, with an odds ratio of 70.50 in a multivariate analysis.

**Table 3:** Logistic regression analysis of risk factors for KRT liberation failure.

Independent variables	Univariate			Multivariate		
	Odds ratio	95% CI	p-value	Odds ratio	95% CI	p-value
Age [years]	0.98	0.94−1.01	0.227	0.97	0.91−1.03	0.312
Male gender	2.07	0.63−6.86	0.232	10.11	1.10−93.13	0.041
PENK ≥250 [pmol/L]	38.25	4.68−312.67	<0.001	70.50	4.48−1110.51	0.002
SCr at last KRT [mg/dL]	1.14	0.79−1.66	0.478	0.82	0.40−1.67	0.582
UO 24 h prior last KRT < 500 mL	4.79	1.71−13.35	0.003	4.38	0.79−24.24	0.091
Diuretic dose 24 h prior last KRT [mg]	1.00	0.99−1.00	0.103	1.00	1.00−1.01	0.894
Liver cirrhosis	7.61	1.53−37.94	0.013	15.57	1.81−133.90	0.012
SOFA Score at baseline	1.02	0.89−1.16	0.783	1.02	0.84−1.24	0.832
Vasopressor support	0.74	0.26−2.13	0.575	1.01	0.14−7.33	0.992

CI confidence interval, KRT kidney replacement therapy, SOFA sequential organ failure assessment, PENK proenkephalin A 119–159, UO urine output.

## Discussion

In this exploratory longitudinal study of a large, unselected ICU cohort, we demonstrate that PENK may offer significant advantages over conventional parameters such as SCr and UO for classifying AKI severity, assessing residual kidney function capacity during KRT, and predicting KRT liberation failure. These findings are relevant to key limitations in current AKI management and may further support the clinical utility of PENK as a physiologically meaningful kidney integrity biomarker, improving standardized care.

The observed AKI incidence (43.2%) and acute KRT requirement (12.6%) in our study were consistent with prior reports [3,10], underscoring the high burden of kidney dysfunction in critical illness. As expected, patients requiring acute KRT presented with greater disease severity, higher mortality, and longer ICU stays. In contrast, patients already on chronic KRT at ICU admission showed lower severity scores, fewer complications such as mechanical ventilation, sepsis, and vasopressor use, and better outcomes, highlighting the diverse outcome trajectories of acute KRT-dependent AKI and chronic KRT.

Among acute KRT patients, 40.9% achieved successful liberation, 24.3% experienced explicit failure requiring re-initiation, and 34.8% either died or were discharged without a completed trial. In patients without any liberation attempts, PENK levels remained elevated – comparable to those with failed liberation – and tended to be higher than in successfully liberated patients, suggesting that PENK mirrors more persistent loss of kidney integrity in alignment with clinical judgment in these patients (Supplementary Figure S2). Both PENK and SCr increased stepwise with KDIGO stage, but only PENK clearly distinguished stage 3 AKI patients with versus without acute KRT requirements, a distinction of prognostic relevance. Furthermore, maximal PENK levels prior to KRT initiation discriminated between acute and chronic KRT, whereas SCr did not, underscoring PENK’s closer reflection of residual function.

Under ongoing KRT, median PENK exhibited reproducible kinetics, increasing progressively regardless of modality (CKRT/SLED or IHD), while SCr predictably declined due to extracorporeal clearance. This divergence is clinically meaningful: SCr reflects clearance efficacy, whereas PENK appears to capture residual endogenous functional capacity and recovery dynamics despite KRT initiation.

In chronic KRT patients, PENK levels were substantially higher compared to acute KRT patients, reflecting the severity and chronicity of kidney dysfunction, and remained stably elevated, while in acute KRT it rose at KRT start and declined around liberation. These reproducible patterns – also recently reported in kidney transplant recipients [26] – support PENK’s validity as a marker of functional integrity. Thus, despite in vitro data suggesting a sieving coefficient near one [29], absolute blood PENK levels seem to remain significantly unaffected by extracorporeal clearance techniques used in ICU patients. A plausible explanation might be that endogenous production, and turnover rates exceed extracorporeal clearance, allowing PENK to retain clinical relevance even under continuous and intermittent clearance conditions. Nevertheless, this hypothesis requires further mechanistic investigation.

While randomized trials in critical care nephrology have primarily addressed the optimal timing for KRT initiation [30], far less evidence guides discontinuation, despite associated risks of unnecessarily prolonged therapy such as infection, bleeding, and hemodynamic instability [31]. In addition, observational studies suggest that failed KRT liberation is associated with increased mortality, although it remains unclear whether this reflects direct harm or is a surrogate for greater disease severity [12]. Still, liberation failure can contribute to fluid overload, metabolic instability and uncertainty in drug dosing, but KRT discontinuation practices are highly variable, with inconsistent decision-making and outcome definitions. Most predictive models and biomarker data come from small, retrospective cohorts and assess parameters only at or after cessation, limiting predictive clinical relevance [31]. Definitions of successful liberation further range from 3 to 60 days without re-initiation, complicating comparability of available studies [31]. A 7-day KRT-free period has increasingly been proposed to define successful liberation [32]; however, this threshold is largely based on expert opinion and has not been prospectively validated. In our study, we selected a 5-day period to capture recovery related to the initial AKI episode while reducing the influence of subsequent clinical events that may independently require re-initiation of KRT. In addition, critically ill patients who remain free of KRT for several days are frequently transferred from the ICU to intermediate care units or external hospitals, making complete follow-up beyond this period increasingly challenging and potentially compromising the completeness and accuracy of outcome assessment.

One of the most salient findings of this study was PENK’s predictive capacity for liberation failure. Levels were significantly higher in patients who failed liberation, both on the day of and 48 h beforehand, while SCr failed to discriminate. ROC analyses confirmed PENK’s superiority, with an AUC of 0.78 around last KRT, that further improved to 0.83 in combination with UO [31,33]. Further, a stepwise combined approach using urine output and PENK levels (Fig. 5) and the presented multivariate analysis, highlight the incremental and additional prognostic value of PENK beyond UO and other relevant covariates, and may provide a framework for independent validation of a decision tool for CKRT liberation.

Two post hoc analyses of the randomized controlled ELAIN and RICH trials evaluated the potential role of PENK in guiding KRT liberation decisions [21,22]. In the post hoc analysis of the ELAIN trial, low PENK concentrations (<89 pmol/L) at KRT initiation or on day 3 after KRT initiation were associated with earlier and successful liberation [21]. In contrast to the ELAIN analysis, the post hoc analysis of the RICH trial found no difference in PENK concentrations at KRT initiation using a 100 pmol/L cut-off, although significant differences were observed on day 3. Nevertheless, urine output >436 mL/day outperformed PENK in predicting successful KRT liberation [22]. These results differ from our findings, as we demonstrate an additional and independent diagnostic value of PENK beyond urine output. This discrepancy is most likely attributable to methodological differences between our study design and the previously reported post-hoc analyses. Unlike fixed timepoint assessments (days 1 and 3 after KRT start) performed in the post hoc analyses of the ELAIN and RICH trial, our study assessed PENK relative to actual discontinuation time point, accounting for variability in disease course and KRT duration. Further, our findings suggest that higher cut-offs may be required to guide discontinuation decisions effectively, since PENK levels were much higher than 100 pmol/L in both patients with liberation failure or success at the time of last KRT. In line with our data, Tichy et al. recently evaluated PENK as a predictor of successful KRT liberation in a small, single-center cohort of 20 patients with cardiac surgery-associated AKI requiring CKRT. [23] Similar to our findings, patients with failed CKRT liberation had significantly higher PENK concentrations at the time of CKRT discontinuation than successfully liberated patients (290 vs. 113 pmol/L), with good discriminatory performance (AUC 0.80). These findings closely mirror our results, in which mean PENK levels at the last KRT session were 238.8 versus 148.4 pmol/L in patients with failed and successful liberation, respectively (AUC 0.78). Whereas Tichy et al. investigated a highly selected cardiac surgery cohort, our study extends these observations to a larger, unselected ICU population and further demonstrates the incremental prognostic value of PENK beyond urine output and other potential covariates of liberation failure.

Although UO is the most extensively studied liberation parameter, its predictive performance is inconsistent (AUC 0.63–0.91), with tested thresholds ranging from 191 to 1700 mL/24 h [31]. In our cohort, UO also showed diagnostic value for predicting liberation failure and further improved diagnostic accuracy in combination with PENK (AUC 0.83). While some studies report diminished predictive performance of UO in the presence of diuretics, others have observed improved discrimination. We saw no predictive value in multivariate analysis for diuretics in our cohort [12,31]. Nevertheless, diuretics remain clinically relevant for fluid management post-KRT, consistent with hypervolemia being the leading indication for re-initiation.

Injury and inflammation biomarkers like NGAL, interleukins, and osteopontin have also been explored for KRT liberation decisions [24,31]. In contrast to functional biomarkers, however, they do not correlate linearly with changes in glomerular filtration or residual kidney function, limiting their utility for KRT liberation in our opinion. Thus, PENK may be the first functional biomarker whose levels remain largely unaffected by KRT modality, while reflecting dynamic functional capacity at the same time.

Finally, although our findings are promising, several limitations should be noted. This was a single-center, exploratory study, and PENK levels were measured in parallel but not used to guide KRT initiation or discontinuation. Thus, interventional trials are needed to validate performance of PENK and to proof, whether algorithms integrating PENK can improve patient-centered outcomes – such as reduced KRT duration, fewer liberation failures, shorter ICU stays, and lower rates of KRT dependency at discharge. In addition, the limited sample size of our KRT liberation cohort resulted in wide confidence intervals for some regression estimates, reflecting uncertainty regarding the exact effect size despite the consistent direction and significance of the observed associations. Further, KRT timing decisions were based on clinical judgment, introducing potential variability; however, all decisions followed institutional standards and were made daily by experienced nephrologists and intensivists in accordance with internal standards, incorporating current evidence from the AKIKI I and II trials [30] (restrictive KRT criteria as illustrated in Supplementary Table S1) and recent data on urine output thresholds for KRT liberation [12], as shown in Supplementary Table S1. Next, although our study suggests that PENK may enable a more nuanced AKI subclassification of severe kidney dysfunction in advanced AKI, we cannot determine whether it represents a more precise filtration marker overall, as measured GFR was not available. Also, PENK measurements were performed on weekdays only. Though analyses of KRT initiation, discontinuation, and liberation were restricted to patients with paired PENK and SCr measurements at the relevant clinical time points, weekday-only sampling may have affected the assessment of reported PENK concentrations across AKI stages. Specifically, peak PENK values may have been missed when occurring during weekends. Lastly, two different dialysis membranes were used across KRT modalities. Although the M100/AN69 membrane has additional adsorptive properties, we observed no systematic differences in PENK trajectories between membrane types (Figure S1). Furthermore, both membranes demonstrate near-complete permeability for small molecules in the size range of PENK, making it unlikely that membrane-specific effects were a major determinant of the observed PENK patterns in our cohort.

To conclude, this study strengthens the evidence that PENK may be a biomarker that surpasses SCr and UO in assessing AKI severity for severe stages, monitoring residual kidney function integrity during KRT, and guiding KRT liberation decisions. Its discriminative power may support its integration into clinical algorithms and predictive models. Prospective multicenter trials are warranted to validate these findings and to assess PENK-guided strategies for standardizing and improving care in ICU patients.

## Authors' contributions

CN and DG conceived the design of the entire study, were responsible for acquisition, analysis, and interpretation of data, and drafted the manuscript. PS assisted with acquisition of data, analysis, interpretation of data and critically revised the manuscript. LB, FK, RH, JG, FU, FCFS, UM, DC, MV, MK, MOF, MZ, CM and MAW contributed to the acquisition and interpretation of the clinical data and critically revised the manuscript. All authors approved the final version of the manuscript for publication.

## Consent for publication

All authors approved the final version of the manuscript for publication.

## Ethics approval and consent to participate

Ethical approval was obtained from the Ethics Committee of the University of Heidelberg (S-714/2021), and the study was registered in the German Clinical Trials Register (DRKS00026776). Patient consent was waived as PENK assessment was integrated into routine diagnostics, imposing no additional burden.

## Funding

The overall project was funded by the Heidelberg Faculty of Medicine and institutional funding from the Departments of Nephrology and Anesthesiology at Heidelberg University Hospital. Assays for quantifying Proenkephalin A 119–159 were funded by SphingoTec. SphingoTec was not involved in the study design, data collection, data analysis and interpretation.

## Data availability statement

The datasets presented in this article are not readily available; however, the data reported are available from the corresponding author on reasonable request. Re-quests to access the datasets should be directed to christian.nusshag@med.uni-heidelberg.de.

## Declaration of competing interest

Christian Nusshag received travel expense reimbursements from SphingoTec. Christian Nusshag and Markus A. Weigand are inventors on a filed patent application related to the use of the PENK assay for early diagnosis/early risk stratification of delayed graft function in kidney transplant recipients. Florian Uhle is an employee and stock option holder of Sphingotec and Inflammatix. All potential, future patent rights have been transferred to SphingoTec GmbH prior to this submission. Christian Nusshag and David Czock received travel expense reimbursements from SphingoTec. SphingoTec was not involved in study design, collection of data, nor in the decision to submit the paper for publication. All other authors of this manuscript have no conflicts of interest to disclose as described by the journal.
