# Supplementary material for: Proenkephalin A for assessing kidney integrity and guiding KRT liberation decisions in critically ill patients
Source: Ann Intensive Care. 2026 Jul 21;16:100113. doi: 10.1016/j.aicoj.2026.100113 (PMC13452179; doi:10.1016/j.aicoj.2026.100113)
Supplement: Supplementary file 1 [file mmc1.pdf]

Supplemental material

Proenkephalin A for Assessing Kidney Integrity and Guiding KRT Liberation Decisions in Critically Ill Patients

David C. Gabriel, Philipp Sauer, Rebecca Happel, Julia Grenz, Louise Benning, Florian Kälble, Florian Uhle, Felix C.F. Schmitt, Uta Merle, David Czock, Markus Verch, Matthias Karck, Mascha O. Fiedler, Martin Zeier, Christian Morath, Markus A. Weigand, Christian Nusshag

Table of contents:

Supplementary tables:

Table S1. Relevant parameters prior first kidney replacement therapy

Table S2. Baseline characteristics stratified by KRT liberation outcome

Supplementary Figures:

Figure S1. A-B Kinetics of PENK and serum creatinine in three individual patients stratified by individual KRT modality.

Figure S2. A-B Comparison of biomarker levels in patients undergoing a KRT liberation attempt with failure or success vs. no liberation trial until discharge or death, and stratified by the timepoint of last KRT (liberation attempt) / last day on ICU (no liberation).

Additional definitions:

Algorithm for defining baseline serum creatinine

Supplemental Tables

Table S1. Relevant parameters prior first kidney replacement therapy

**Parameters at KRT start total**	
Serum creatinine – [mg/dL]	2.42 (1.60-3.46)
PENK – [pmol/L]	106.9 (73.9-163.9)
Urea – [mg/dL]	109 (66-170)
Serum potassium – [mmol/L]	5.3 (4.85-5.75)
pH	7.32 (7.26-7.37)
Cumulative fluid balance – [mL]	3356 (1674-6844)
**Parameters at KRT start stratified by indication (N=181)**	**Start KRT**
**Hypervolemia (N=109)**	
Cumulative fluid balance [mL]	3827 (2357-7176)
PENK [pmol/L]	118.4 (91.3-179.8)
Serum creatinine [mg/dL]	2.15 (1.32-3.02)
**Uraemia (N=32)**	
Urea [mg/dL]	214 (204-248)
PENK [mg/dL]	142.9 (69.6-209.3)
Serum creatinine [mg/dL]	4.04 (2.56-4.9)
**Hyperkalaemia (N=31)**	
Serum potassium [mmol/L]	6.12 (5.88-6.28)
PENK [pmol/L]	88.3 (61.0-106.4)
Serum creatinine [mg/dL]	2.61 (1.75-3.47)
**Metabolic acidosis (N=9)**	
pH	7.17 (7.13-7.2)
PENK [pmol/L]	102.0 (74.1-124.0)
Serum creatinine [mg/dL]	2.78 (1.71-3.30)

Laboratory values and parameters that led to the decision to undergo dialysis of the patients with acute KRT. KRT kidney replacement therapy, PENK proenkephalin.

Table S2. Baseline characteristics stratified by KRT liberation outcome

**Demographics**	**Liberation Success**	**Liberation failure**	**p-value**
Age – yr	67 (55-73)	58 (51-70)	0.120
Female sex – no. (%)	10 (27.0)	5 (15.2)	0.227
Body-Mass-Index – kg/m^2	28.1 (23.0-31.8)	26.0 (22.5-35.3)	0.787
Baseline serum creatinine prior ICU (w/o ESRD) – mg/dL (IQR)[α]	1.11 (0.87-1.33)	0.91 (0.66-1.30)	0.193
**Kidney parameters and ICU Scores on admission**			
PENK – pmol/L (IQR)	95.3 (48.2-131.6)	93.4 (58.1-147.2)	0.555
Serum creatinine	1.75 (1.06-2.85)	2.07 (1.23-3.00)	0394
Urea – mg/dL (IQR)	62 (48-88)	83 (62-123)	0.043
Oliguria or anuria – no. (%) ǂ	8 (21.6)	15 (45.5)	0.034
SOFA score[β]	11 (9-13)	13 (10-15.5)	0.186
SAPS II value[γ]	59 (38-73)	66 (45-80)	0.251
APACHE II score[δ]	28 (20-34)	30 (24-36)	0.494
**Preexisting comorbidities – no. (%)**			
Chronic kidney disease (eGFR <60 mL/min/1,73m^2)	23 (62.2)	16 (48.5)	0.250
End-stage kidney disease	0 (0.0)	0 (0.0)	>0.999
Arterial Hypertension	25 (67.6)	19 (57.6)	0.388
Coronary artery disease	13 (35.1)	6 (18.2)	0.111
Congestive heart failure	11 (29.7)	2 (6.1)	0.011
Aortic aneurysm	7 (18.9)	2 (6.1)	0.109
Diabetes mellitus	9 (24.3)	9 (27.3)	0.778
Liver cirrhosis	2 (5.4)	10 (30.3)	0.006
Malignant disease	7 (18.9)	5 (15.2)	0.676
**Outcomes**			
Mechanical ventilation – no. (%)	33 (89.2)	26 (78.8)	0.229
Sepsis – no. (%)	18 (48.6)	23 (69.7)	0.074
Septic shock – no. (%)	18 (48.6)	23 (69.7)	0.074
Vasopressor support – no. (%)	36 (97.3)	30 (90.9)	0.250
Maximum serum creatinine – mg/dL (IQR)	3.52 (2.60-4.49)	5.14 (3.63-6.82)	<0.001
Maximum PENK – pmol/L (IQR)	211.7 (135.4-286.1)	399.2 (263.4-536.3)	<0.001
Length of ICU stay – days	41 (19-64)	46 (34-74)	0.104
Hospital mortality (%)	11 (29.7)	10 (30.3)	0.467

* Values are represented as median plus (interquartile range). Listed are data from adult patients who have at least one PENK value. AKI denotes acute kidney injury, eGFR CKD-Epi estimated glomerular filtration rate chronic kidney disease epidemiology collaboration, IQR interquartile range.

α Baseline serum creatinine was acquired for all patients without end-stage renal disease.

β Scores on the Sequential Organ Failure Assessment (SOFA) range from 0 to 24, with larger values indicating greater disease severity and mortality.

γ Results for the Simplified Acute Physiology Score (SAPS) II range from 0 to 163, with higher scores indicating greater disease severity and mortality.

δ Results for the Acute Physiology And Chronic Health Evaluation (APACHE) II score range from 0 to 71, with higher scores indicating greater disease severity and mortality.

ǂ Oliguria was defined as urinary output < 500 ml within a 24-hour period.

Supplemental Figures

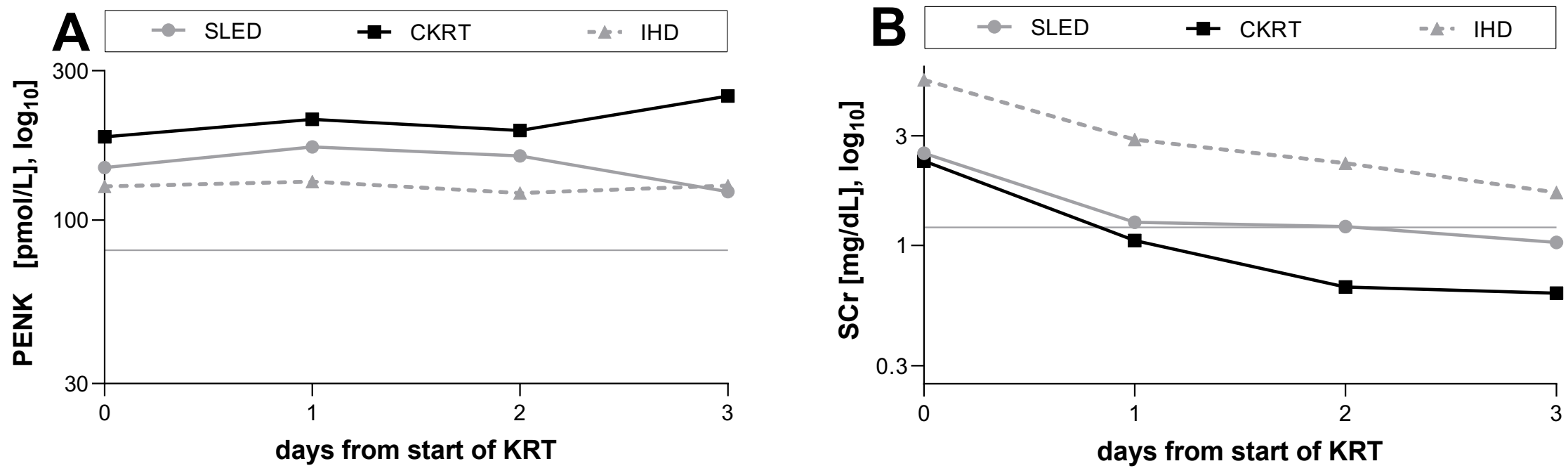

Figure S1. A-B Kinetics of PENK and serum creatinine in three individual patients stratified by individual KRT modality. CKRT continuous kidney replacement therapy (M100/AN69), IHD intermittent haemodialysis (Fx50), KRT kidney replacement therapy, SLED Sustained low efficiency dialysis (Fx50),

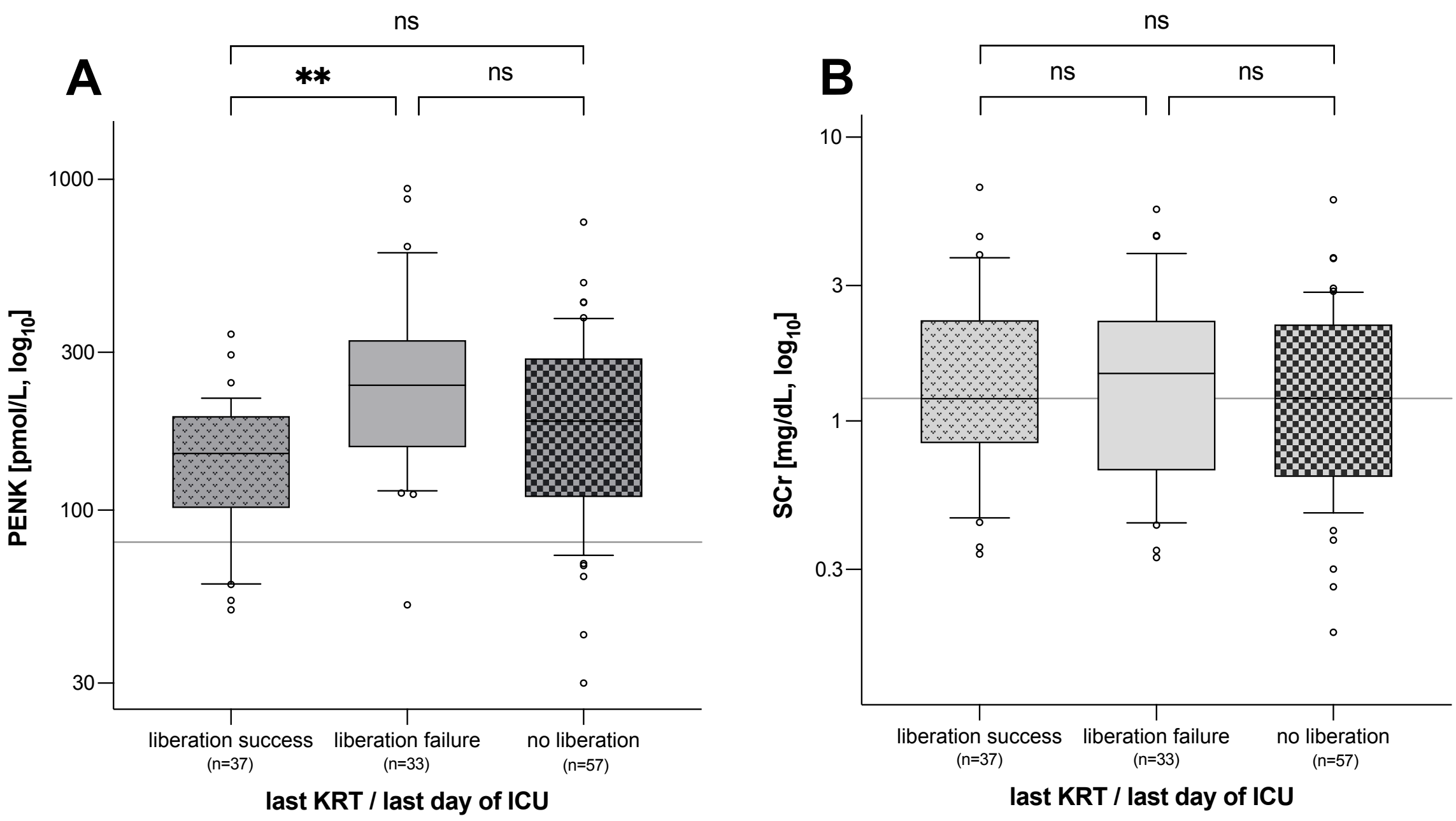

Figure S2. A-B Comparison of biomarker levels in patients undergoing a KRT liberation attempt with failure or success vs. no liberation trial until discharge or death, and stratified by the timepoint of last KRT (liberation attempt) / last day on ICU (no liberation). PENK proenkephalin, KRT kidney replacement therapy, SCr serum creatinine. Gray lines indicate upper normal range. Statistical significance is indicated as follows: ns, $P \geq 0.05$; $P < 0.05$ (*); $P < 0.01$ (**); $P < 0.001$ (***); $P < 0.0001$ (****).

Additional definitions

<u>Algorithm for defining baseline serum creatinine:</u>

Definition of baseline serum creatinine (SCr) in descending order:

1. the mean of SCr values from three months to eight days prior to hospital admission was calculated and established as the baseline creatinine.
2. the nadir from seven days to one day before hospitalization was selected.
3. the SCr value measured on the day of hospitalization was determined as the baseline creatinine.
4. the baseline SCr was selected from values measured within one year prior to hospitalization. Preferably, at least three values marked a steady plateau that was not in an acute phase of a disease.
5. the eGFR-MDRD formula was rearranged for SCr and back-calculated.
